# Supplementary material for: Causal interplay between lactose intolerance and gut microbiota: a combined bidirectional Mendelian randomization and in vivo validation study
Source: Front Nutr. 2026 Jun 1;13:1803337. doi: 10.3389/fnut.2026.1803337 (PMC13265576; doi:10.3389/fnut.2026.1803337)
Supplement: Supplementary file 1 [file Data_Sheet_1.zip › supplementary materials/Forward/forest plot/ebi-a-GCST90027817.finngen_R12_E4_LACTONAS.pdf]

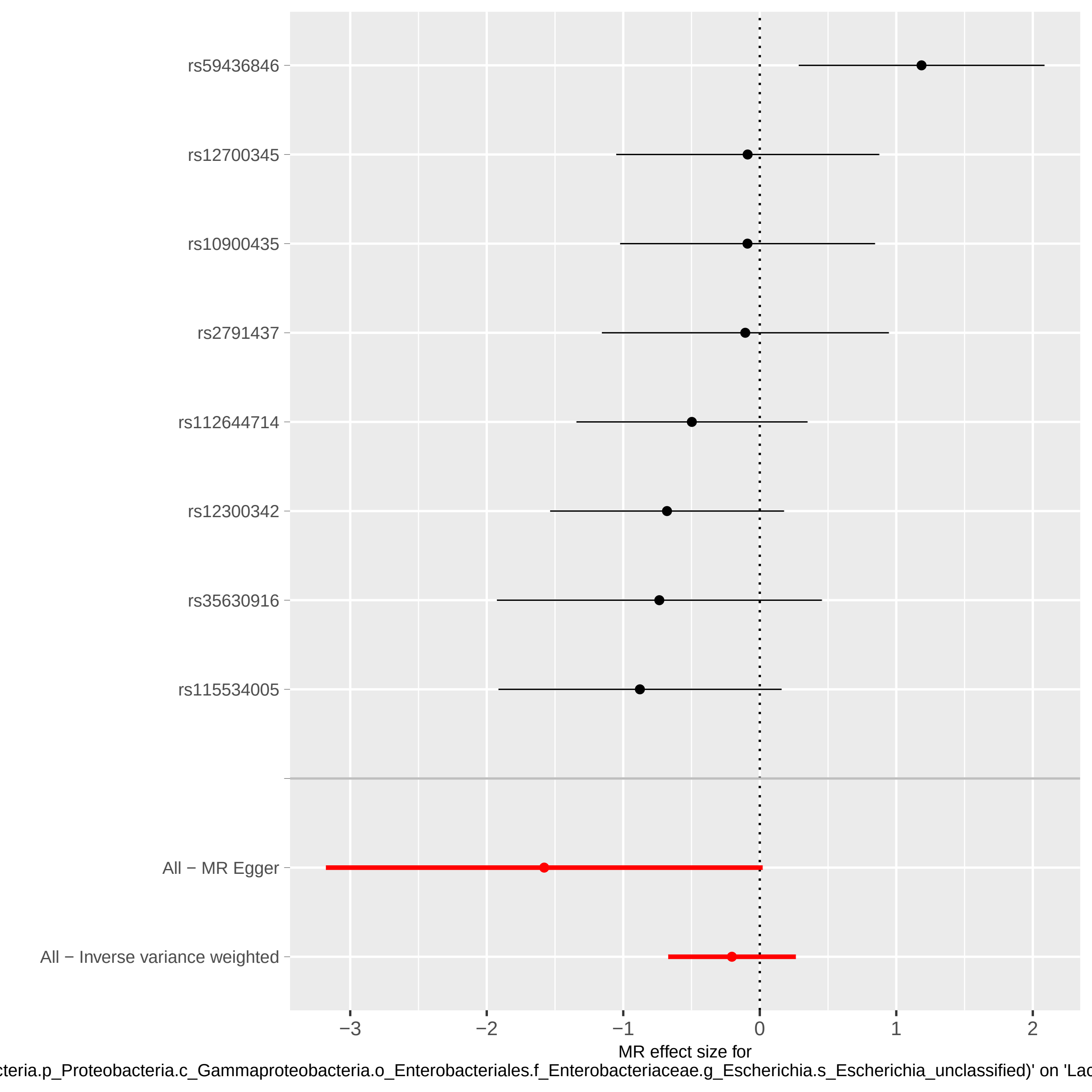
rs59436846
rs12700345
rs10900435
rs2791437
rs112644714
rs12300342
rs35630916
rs115534005
All − MR Egger
All − Inverse variance weighted
−3
−2
−1
0
1
2
MR effect size for
cteria.p_Proteobacteria.c_Gammaproteobacteria.o_Enterobacteriales.f_Enterobacteriaceae.g_Escherichia.s_Escherichia_unclassified)' on 'Lac
